# Naturally Prefabricated Marine Biomaterials: Isolation and Applications of Flat Chitinous 3D Scaffolds from *Ianthella labyrinthus* (Demospongiae: Verongiida)

**DOI:** 10.3390/ijms20205105

**Published:** 2019-10-15

**Authors:** Mario Schubert, Björn Binnewerg, Alona Voronkina, Lyubov Muzychka, Marcin Wysokowski, Iaroslav Petrenko, Valentine Kovalchuk, Mikhail Tsurkan, Rajko Martinovic, Nicole Bechmann, Viatcheslav N. Ivanenko, Andriy Fursov, Oleg B. Smolii, Jane Fromont, Yvonne Joseph, Stefan R. Bornstein, Marco Giovine, Dirk Erpenbeck, Kaomei Guan, Hermann Ehrlich

**Affiliations:** 1 Institute of Pharmacology and Toxicology, Technische Universität Dresden, 01307 Dresden, Germany; mario.schubert1@tu-dresden.de (M.S.); bjoern.binnewerg@tu-dresden.de (B.B.); 2 Department of Pharmacy, National Pirogov Memorial Medical University, Vinnytsya, 21018 Vinnytsia, Ukraine; algol2808@gmail.com; 3 V.P Kukhar Institute of Bioorganic Chemistry and Petrochemistry, National Academy of Science of Ukraine, Murmanska Str. 1, 02094 Kyiv, Ukraine; lmuzychka@rambler.ru (L.M.); smolii@bpci.kiev.ua (O.B.S.); 4 Faculty of Chemical Technology, Institute of Chemical Technology and Engineering, Poznan University of Technology, Berdychowo 4, 60-965 Poznan, Poland; marcin.wysokowski@put.poznan.pl; 5 Institute of Electronics and Sensor Materials, TU Bergakademie Freiberg, Gustav-Zeuner str. 3, 09599 Freiberg, Germany; iaroslavpetrenko@gmail.com (I.P.); andriyfur@gmail.com (A.F.); yvonne.joseph@esm.tu-freiberg.de (Y.J.); 6 Department of Microbiology, National Pirogov Memorial Medical University, Vinnytsya, 21018 Vinnytsia, Ukraine; valentinkovalchuk2015@gmail.com; 7 Leibniz Institute of Polymer Research Dresden, 01069 Dresden, Germany; tsurkan@ipfdd.de; 8 Institute of Marine Biology, University of Montenegro, 85330 Kotor, Montenegro; rajko.mar@ucg.ac.me; 9 Institute of Clinical Chemistry and Laboratory Medicine, University Hospital Carl Gustav Carus, Faculty of Medicine Carl Gustav Carus, Technische Universität Dresden, 01307 Dresden, Germany; nicole.bechmann@uniklinikum-dresden.de; 10 Department of Invertebrate Zoology, Biological Faculty, Lomonosov Moscow State University, 119992 Moscow, Russia; ivanenko.slava@gmail.com; 11 Aquatic Zoology Department, Western Australian Museum, Locked Bag 49, Welshpool DC, WA 6986, Australia; Jane.Fromont@museum.wa.gov.au; 12 Department of Internal Medicine III, University Hospital Carl Gustav Carus, Technische Universität Dresden, 01307 Dresden, Germany; Stefan.Bornstein@uniklinikum-dresden.de; 13 Diabetes and Nutritional Sciences Division, King’s College London, London WC2R 2LS, UK; 14 Department of Sciences of Earth, Environment and Life, University of Genoa, Corso Europa 26, 16132 Genova, Italy; mgiovine@unige.it; 15 Department of Earth and Environmental Sciences & GeoBio-Center, Ludwig-Maximilians-Universität München, Richard-Wagner-Str. 10, 80333 Munich, Germany; erpenbeck@lmu.de

**Keywords:** chitin, scaffold, marine sponges, cardiomyocytes, tissue engineering, hemostats, wound dressing

## Abstract

Marine sponges remain representative of a unique source of renewable biological materials. The demosponges of the family Ianthellidae possess chitin-based skeletons with high biomimetic potential. These three-dimensional (3D) constructs can potentially be used in tissue engineering and regenerative medicine. In this study, we focus our attention, for the first time, on the marine sponge *Ianthella labyrinthus* Bergquist & Kelly-Borges, 1995 (Demospongiae: Verongida: Ianthellidae) as a novel potential source of naturally prestructured bandage-like 3D scaffolds which can be isolated simultaneously with biologically active bromotyrosines. Specifically, translucent and elastic flat chitinous scaffolds have been obtained after bromotyrosine extraction and chemical treatments of the sponge skeleton with alternate alkaline and acidic solutions. For the first time, cardiomyocytes differentiated from human induced pluripotent stem cells (iPSC-CMs) have been used to test the suitability of *I. labyrinthus* chitinous skeleton as ready-to-use scaffold for their cell culture. Results reveal a comparable attachment and growth on isolated chitin-skeleton, compared to scaffolds coated with extracellular matrix mimetic Geltrex^®^. Thus, the natural, unmodified *I. labyrinthus* cleaned sponge skeleton can be used to culture iPSC-CMs and 3D tissue engineering. In addition, *I. labyrinthus* chitin-based scaffolds demonstrate strong and efficient capability to absorb blood deep into the microtubes due to their excellent capillary effect. These findings are suggestive of the future development of new sponge chitin-based absorbable hemostats as alternatives to already well recognized cellulose-based fabrics.

## 1. Introduction

During the last 50 years, marine sponges belonging to the class Demospongiae (Porifera) have been recognized as a high potential source of bioactive secondary metabolites (for review, see [1,2]), as well as biological materials of proteinaceous [3] and polysaccharide [4,5,6,7,8] origin. Broad diversity of secondary metabolites, mostly alkaloids and peptides, have been studied as potential antibacterial, antiviral, antifungal, and anticancer agents [9,10]. Biological materials such as structural collagenous proteinaceous spongin and aminopolysaccharide chitin have found applications in technology [3,11,12], extreme biomimetics [13,14,15,16,17,18], electrochemistry [19], and tissue engineering [20,21,22,23,24]. Thus, demosponges continue to be productive organisms for investigations of both marine pharmacology and biologically inspired materials science. A critical factor is the ability of demosponges to regenerate tissues and skeletons, and to grow under marine farming conditions [25,26] or under biotechnological sustainable biomass production based on cell culture [27]. Consequently, the recent trend in practical applications of demosponges is based on the development of approaches where both secondary metabolites and biomaterials can be extracted simultaneously. Until now, selected demosponges have been extracted with diverse organic reagents to isolate secondary metabolites, while the tissue and skeletal components have not been utilized.

Since 2007, scientific interest in simultaneous extraction strategies of demosponges has focused on sponge representatives of the order Verongiida within the class Demospongiae [4]. These sponges are known to synthesize naturally prefabricated three-dimensional (3D) chitin scaffolds [4,5,6,8,28,29,30,31] and contain highly biologically active bromotyrosines [2,32,33,34]. Biological reasons to produce bromotyrosines, which are localized within spherulocytes [35,36] in skeletal chitinous fibers of verongiids, are likely to be related to the inhibitory activity of these metabolites against microbial chitinases [37]. Numerous experimental studies dedicated to determining the multitarget activities of selected bromotyrosines have also confirmed antiviral [38], antibacterial [39], antiparasitic [40], anti-inflammatory [41], antitumor [2,10], and enzyme-inhibitory and epigenetic [42] properties.

**Figure 1:**
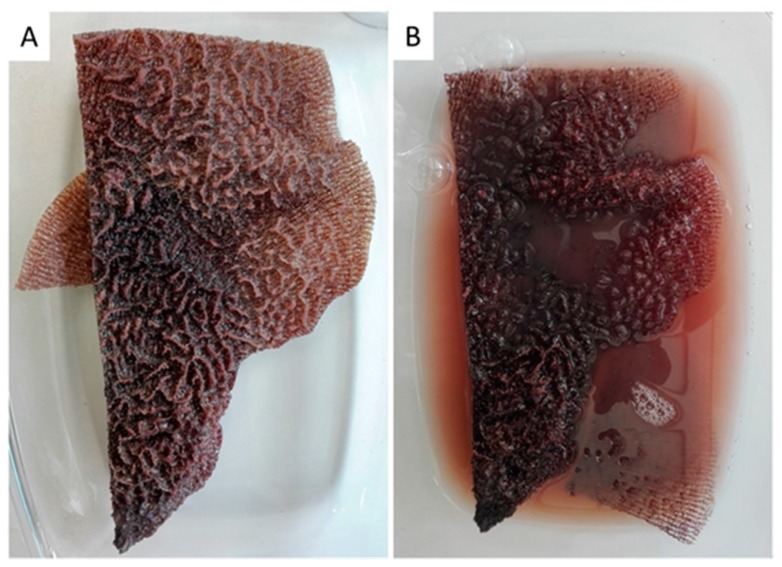
A fragment (20 × 10 cm) of the demosponge *Ianthella labyrinthus* after sampling (**A**) shows labyrinth-like surface morphology (**B**). It loses pigmentation immediately after placement into distillated water due to osmotic shock.

Skeletons of all verongiid demosponges studied so far (see overview [6,8]) are made of fibrous, tubular, anastomozing chitinous 3D networks that are cylinder-like (i.e., sponges belonging to the family Aplysinidae, [4,5,8,31]) or fan-shaped and flat (family Ianthellidae, [30,32,43]). The demosponges of the Ianthellidae (Figure 1) have flat (up to 5 mm thick), naturally prefabricated chitin-based skeletons with high biomimetic potential due to their ability to regenerate chitinous tissues in vivo [43]. We suggest that these sponges are suitable as ready-to-use constructs to replace damaged skin fragments, or as alternatives for wound dressing, including application after plastic surgery treatments.

In addition, we have reported previously that the chitinous skeletal structures of *Ianthella* sponges [30] have applications for tissue engineering of selected human bone marrow-derived mesenchymal stromal cells (hBMSCs) and human dermal MSCs [23,24].

In this study, for the first time, we focus our attention on *Ianthella labyrinthus* Bergquist & Kelly-Borges, 1995 (Demospongiae: Verongiida: Ianthellidae) as a novel potential source for isolation of naturally prestructured bandage-like 3D scaffolds which can be isolated simultaneously with bromotyrosines. Also for the first time, we choose human induced pluripotent stem cells (iPSC-CMs) to test the suitability of the *I. labyrinthus* chitinous scaffold for cell culture. The first experiments testing the ability of sponge chitin to absorb blood have also been carried out.

## 2. Results and Discussion

### 2.1. Isolation of 3D Chitin Scaffolds

**Figure 2:**
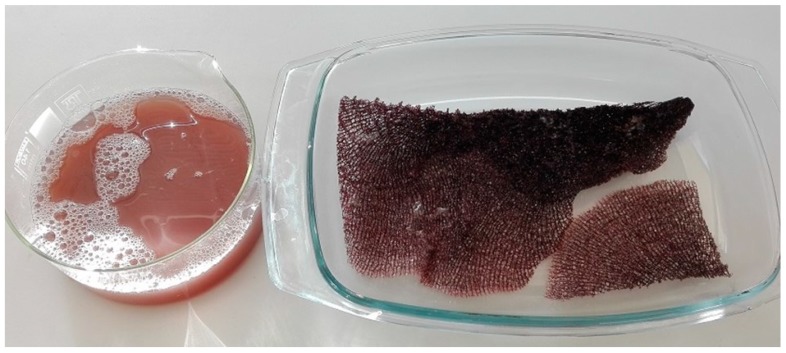
Typical flat mesh-like architecture of *I. labyrinthus*, mechanically rigid and pigmented, visible after 24 h insertion in distilled water at room temperature. Pigments and cell debris obtained due to osmotic shock have been partially removed from the skeleton using distilled water.

**Figure 3:**
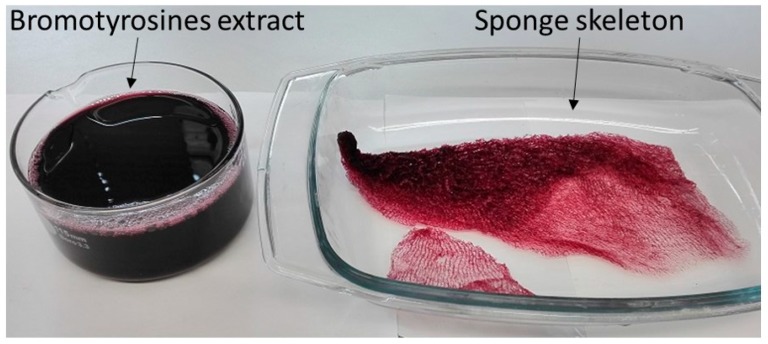
Bromotyrosine-containing extract and bromotyrosine-containing flexible skeleton of *I. labyrinthus* after 48 h treatment with 2.5 M NaOH at 37 °C.

The body architecture of most representatives of the Ianthellidae is characterized by a fan-shaped form [43,44,45]. In these sponges, tissues are localized on and within a mechanically rigid and dark-reddish pigmented meshwork which is produced by interconnected microtubular chitinous fibers (for details, see [30]). This mesh-like morphology becomes visible after insertion of the sponges (Figure 1 and Figure 2) in distillated water, or following treatments with 2.5 M NaOH solution at 37 °C (Figure 3). Both kinds of extracts contain bromotyrosines and can be used for isolation, identification, and possible applications of these biologically active compounds [46,47,48] in separate future research.

**Figure 4:**
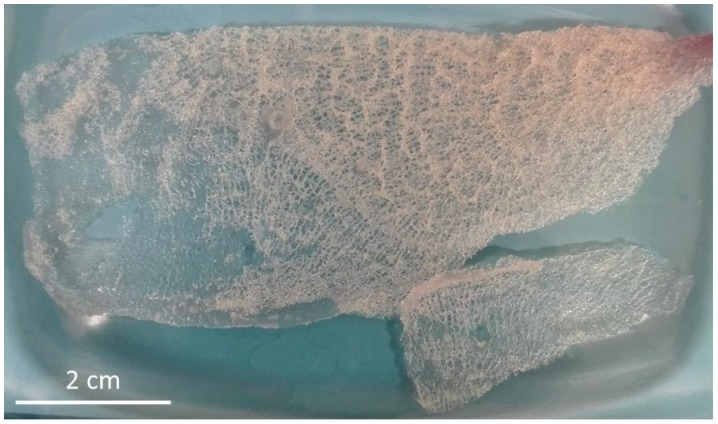
Translucent and mechanically flexible (see also Figure 5) chitinous skeleton of *I. labyrinthus* isolated after alternating use of acid and alkali treatments for 72 h at 37 °C.

**Figure 5:**
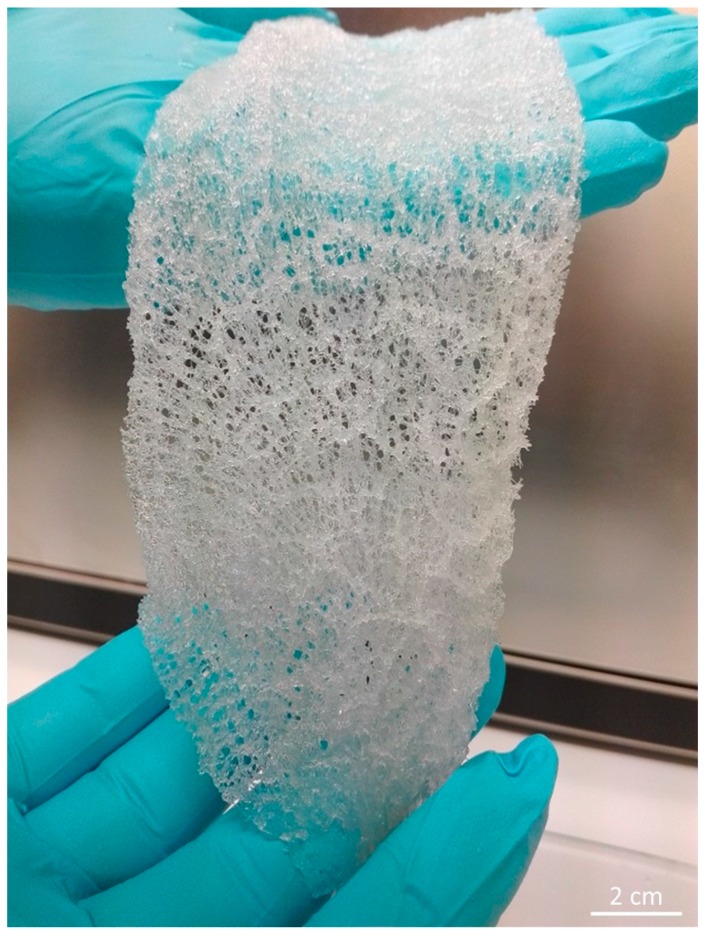
Ready-to-use, flat 3D chitinous scaffold isolated from *I. labyrinthus* remains porous and is mechanically strong enough to be manually manipulated.

**Figure 6:**
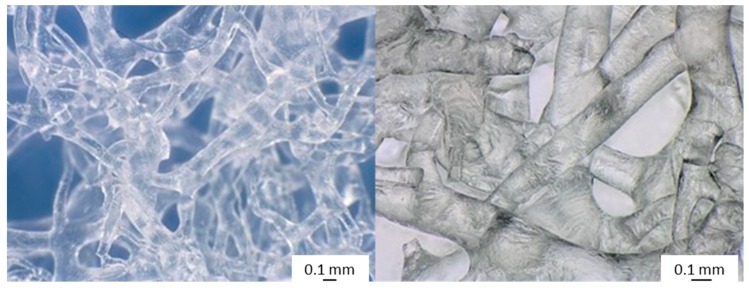
Chitinous 3D scaffolds isolated from *I. labyrinthus* showing a microtubular, interconnected meshwork. These microtubes are able to absorb water, as well as media, for cultivation of cells due to capillary forces. The porous space is filled with air.

Translucent, elastic, flat scaffolds (Figure 4 and Figure 5) can be obtained from the cell-free skeleton of *I. labyrinthus* after alternating treatments described below. These unique gauze fabric-like natural constructs are able to be manually manipulated and assume the shape of corresponding hard surfaces on which they are placed. This feature, together with an excellent ability to swell with diverse liquids (Figure 6) in a few seconds (due to a capillary effect [8]), as well as structural stability after sterilization up to 200 °C [49], may allow for practical application of such scaffolds in regenerative medicine using the principles of tissue engineering.

### 2.2. 3D Chitin Scaffold from I. labyrinthus as Model System for Tissue Engineering of Cardiomyocytes

**Figure 7:**
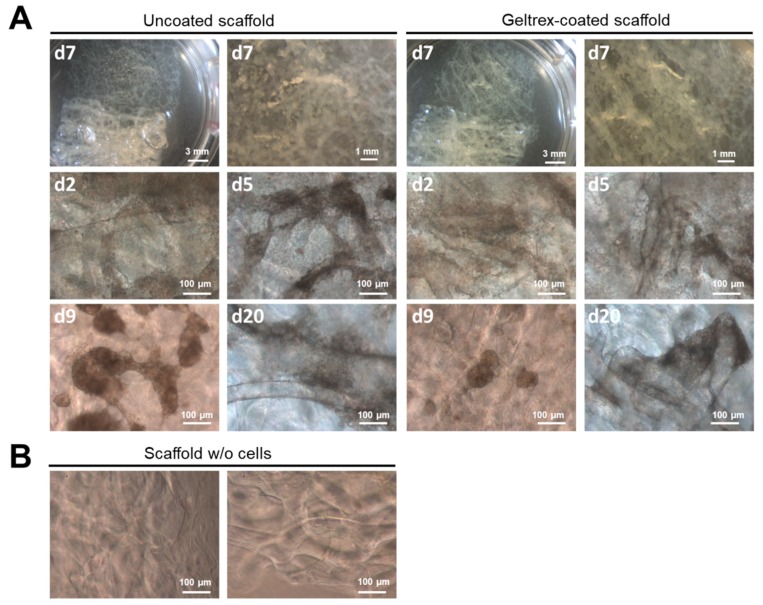
Evaluation of *I. labyrinthus* structures to culture iPSC-derived cardiomyocytes. (**A**) Schematic image of sponge structures cultured in transwell plates with low medium levels, 8-week-old iPSC-CMs were supplied by medium absorbed by the sponge structure and cultured in uncoated and Geltrex^®^-coated sponge scaffolds for 20 days. (**B**) Microscopic images of sponge scaffold. iPSC-CMs were visualized using phase contrast microscopy. Images from two experiments performed using iPSC-CMs from different healthy donors. See also respective video files in the Supplementary Materials.

After positive results with the chitinous scaffolds of *I. basta* for cultivation of diverse hBMSCs and human dermal MSCs [23,24], we investigated the flat scaffolds of *I. labyrinthus* with respect to their application for growth of human iPSC-CMs. The aim of this research was to culture iPSC-derived cardiac muscle cells, termed cardiomyocytes, using 3D sponge chitin-based scaffolds. As reported previously [50], fetal or neonatal rat cardiomyocytes were used for 3D tissue substitutes and were able to integrate structurally and functionally with host myocardial tissue when transplanted into injured myocardium. Consequently, we suggest that 3D chitin scaffolds may act as tissue mimicking geometrical constructs [51], and could be used as alternative models to investigate cardiac metabolism and cardiac remodeling and regeneration [52]. Thus, we chose iPSC-CMs to test the suitability of the isolated chitinous sponge scaffolds for cell culture. Contractile behavior over a longer culture period requires a stable adhesion to the surface material. iPSC-CMs spontaneously contract due to differentiation, leading to different populations of cardiac cells, including a small number of pacemaker-like cells [53]. The cultivation of iPSC-CMs was investigated on pure chitin scaffolds, in comparison to material that was pre-coated with the extracellular matrix mimetic Geltrex^®^, which is used in standard iPSC-CM culture approaches using commercial plastic plates (Figure 7). Documentation of cell attachment and behavior using these materials is challenging, because of the 3D structure.

After seeding, iPSC-CMs adhered on the coated, as well as uncoated, scaffolds. Microscopic documentation revealed beating iPSC-CMs 24 h after seeding, which is comparable to the behavior observed using standard commercially available culture materials (Supplementary Materials Video 1). Over the culture period of 20 days, iPSC-CMs showed stable contraction behavior and formed contracting cell clusters, which connected different chitin fibers (Figure 7). Contractions of these clusters and deformation of the material were visible even using low magnification (Figure 7 and Supplementary Materials Video 1).

**Figure 8:**
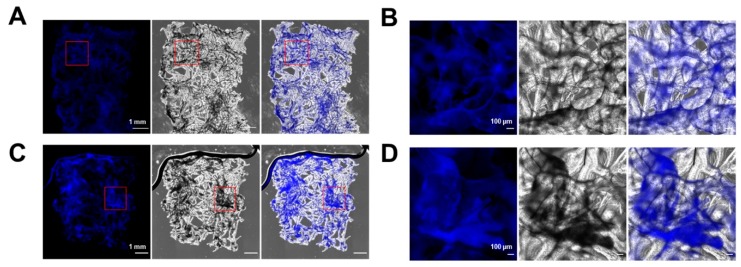
Distribution of iPSC-CMs on Geltrex^®^-coated versus uncoated chitin scaffolds of *I. labyrinthus.* Representative images of 1-month-old iPSC-CMs that were cultured in chitin scaffolds for 20 days. Cell nuclei were stained with Hoechst33342, blue. (**A**,**C**) Overview images of Geltrex-coated (**A**) and uncoated (**C**) chitin scaffolds. (**B**,**D**) Higher magnification of areas indicated in (**A**) and (**C**), respectively.

To analyze the distribution, structural organization, and proliferation of iPSC-CMs, α-actinin, Ki-67, and cell nuclei were stained and visualized using fluorescence microscopy. To compare the distribution of iPSC-CMs on natural and Geltrex^®^-coated scaffolds, phase contrast images were combined via multi-dimensional detection mode (images obtained with 4× magnification, XY stitching). To specify the signals to attached cells, Hoechst33342 signals were detected, because the intensity of this dye can be detected at lower magnifications (Figure 8). The overview images of the sponge scaffolds illustrate the distribution of the iPSC-CMs over the complete chitin structures in Geltrex^®^-coated, as well as in uncoated, sponge scaffolds.

**Figure 9:**
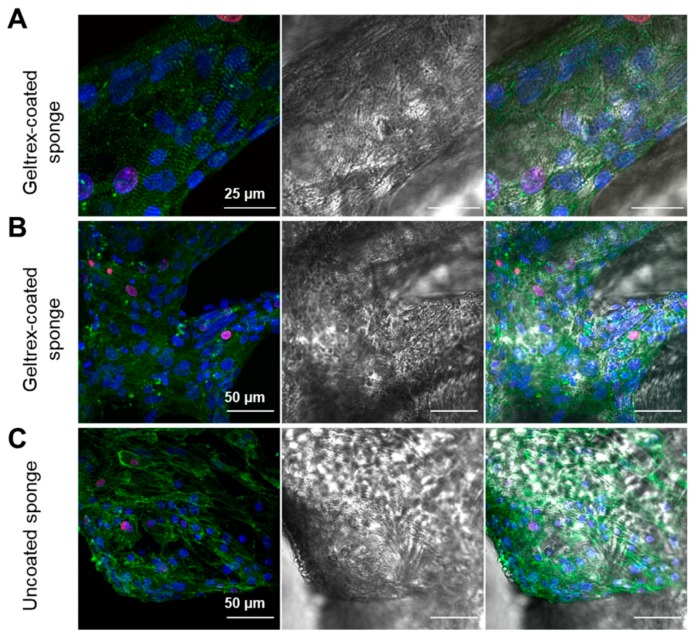
Staining of α-actinin, Ki-67, and cell nuclei to document cell alignment and proliferation of iPSC-CMs on *I. labyrinthus* scaffolds. Left panel shows fluorescence channels of α-actinin (green), Ki-67 (magenta), and nuclei (blue), middle panel represents brightfield image, and right panel overlay of brightfield and fluorescence channels. (**A**) iPSC-CMs on Geltrex^®^-coated scaffold representing sarcomere structures of cells attached to a chitin fiber and the presence of a minor population of Ki-67 positive cells. (**B**,**C**) Representative images of iPSC-CMs on Geltrex^®^-coated and uncoated sponge scaffolds.

Z-stacking of single images was performed to illustrate cell attachment in the 3D scaffold. α-actinin is a structural marker for the detection of sarcomere units in cardiomyocytes (Figure 9A). We can clearly observe that iPSC-CMs oriented parallel to the long axis of the chitin scaffolds, as demonstrated by the repeatable, regular positioning of the sarcomeres in iPSC-CMs attached to the chitin scaffolds (Figure 9A). With respect to this point, it is important to explain that stretching forces [54] were characterized as important stimuli to induce the structural and functional maturation of iPSC-CMs—for example, in engineered heart muscles (EHMs) [55]. Future studies should be focused on the quantitative analysis of cardiomyocyte alignment on the chitin scaffolds in comparison to two-dimensional (2D) monolayer culture of iPSC-CMs and to 3D EHMs as described previously [55]. A reliable, robust quantification method is required when alignment is determined based on structural markers [56,57].

Proliferation of the iPSC-CMs on the sponge scaffolds was investigated using the marker protein Ki-67, which has been used to quantify CM proliferation in different studies [58,59]. Proliferating cells are indicated by the colocalization of Ki-67 with cell nuclei (Figure 9A). Relative quantification was performed by cell counting based on different images from two independent experiments (Figure 9B,C, total of >300 cells per experiment). The fraction of positive cells was determined with 4.7 ± 0.7% Geltrex^®^-coated and 7.1 ± 1.8% in pure, uncoated scaffolds. These results are comparable to other reports, which demonstrate approximately 5% Ki-67 positive cells in a population of 1-month-old iPSC-CMs [58,59].

**Figure 10:**
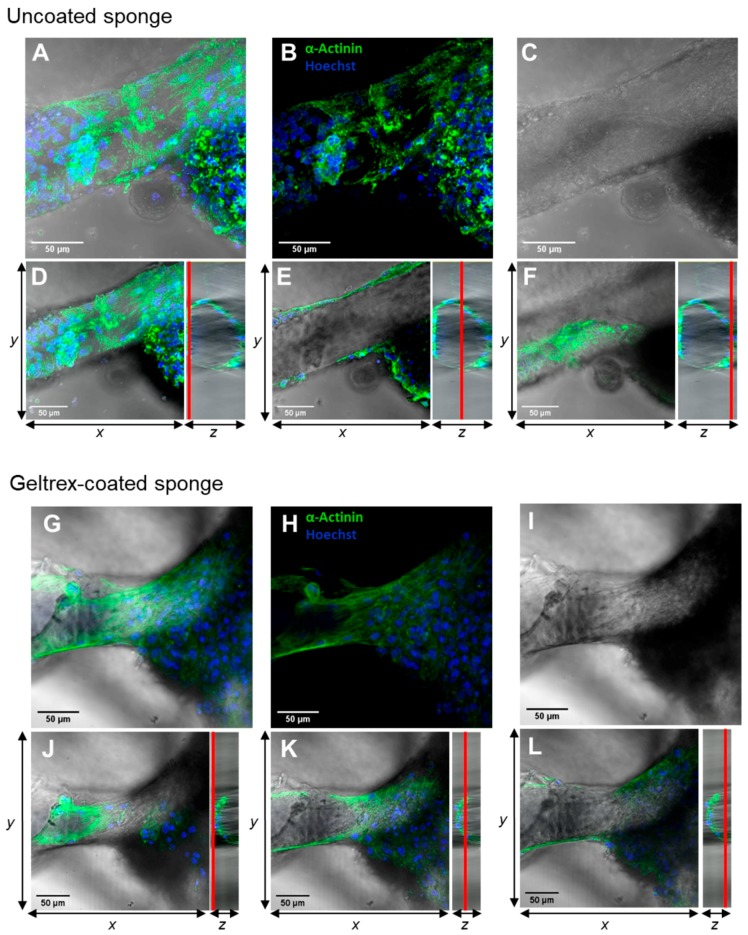
Documentation of iPSC-CM attachment using fluorescence microscopy. Images of iPSC-CMs on *I. labyrinthus* chitin scaffolds (marked as “sponge”) have been obtained as z-stacks of 90–250 images using confocal laser scanning microscopy. (**A**–**C**,**G**–**I**) Combined images of all z-stacks in this area. (**D**–**F**,**J**–**L**) Combined images of different z-positions indicated by the red line in yz-plots.

Furthermore, an orthogonal view was projected from stacks of 90–250 images in different z-orientations (50–150 µm height) to characterize iPSC-CM attachment around chitin fibers in 3D (Figure 10). The overall signals (sum of all images) in Figure 10A–E demonstrate the attachment of iPSC-CMs along a chitin fiber, based on localization of α-actinin and cell nuclei signals. However, analysis of specific regions in defined z-positions, indicated by red lines in YZ-projections, demonstrate that iPSC-CMs attached to the upper surface (Figure 10D), the side areas (Figure 10E), as well as the bottom of the chitin fibers (Figure 10F).

The focus of these initial experiments was to investigate the biocompatibility of the prefabricated *I. labyrinthus* chitinous scaffolds to culture iPSC-CMs. Definitively, further studies may be performed to examine the cell adhesion rates of iPSC-CMs using these natural constructs, especially on large-size matrices. Taken together, our findings provide the basis for further studies to investigate the use of *I. labyrinthus* scaffolds in advanced iPSC-CM culture models as engineered heart muscles (EHMs, [55]).

Beyond the scope of this study, the investigation of further aspects, especially with respect to the structural and functional maturation of iPSC-CMs, as well as the culture in combination with cardiac fibroblasts, are inevitable to clarify the potential of *I. labyrinthus* scaffolds for cardiac tissue engineering. These aspects will further enable the determination of quantitative data and thus the comparison of chitin scaffolds to other materials described for the cultivation of iPSC-CMs and cardiac tissue engineering, including using decellularized hearts (reviewed by [60]), plant scaffolds [61], and hydrogels [62]. Previous studies further highlight the lack of a vascular network as a major limitation of current tissue engineering approaches, because the oxygen diffusion limit in tissues is approximately 100–200 µm [61], and recent studies have tried to address this issue using porous scaffolds [63]. Here, it will be interesting to study whether the mesoporous structure of the chitin scaffolds may have positive effects on cell survival in bigger EHMs and offer the possibility to integrate endothelial cells in an advanced model system, as performed for decellularized leaf structures [61].

### 2.3. 3D Chitin Scaffolds of Poriferan Origin as Alternative Gauze Fabrics

**Figure 11:**
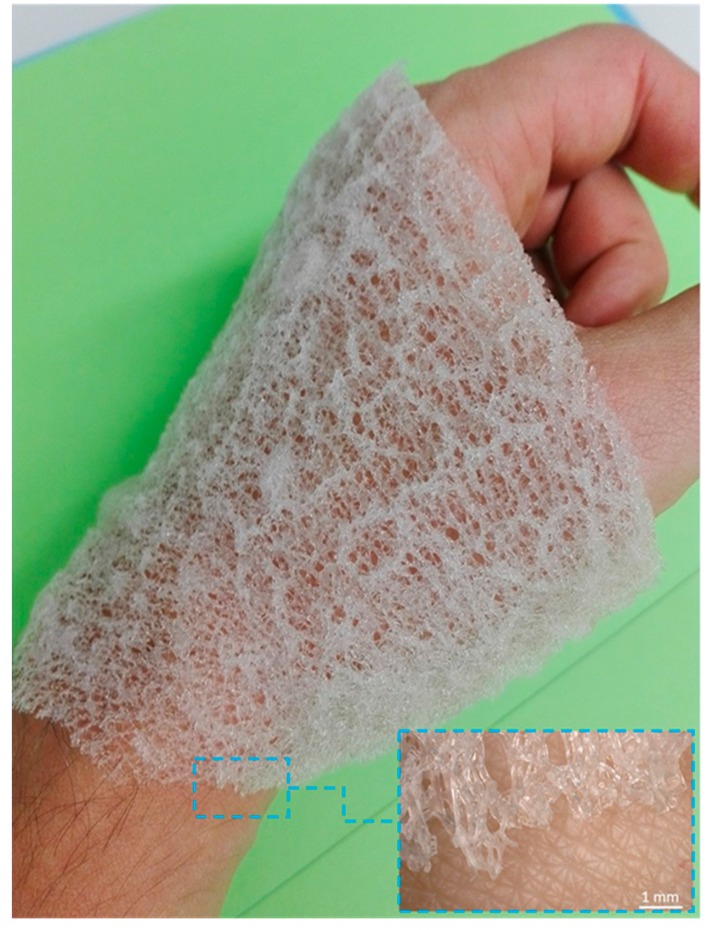
Gauze fabric-like morphology of chitinous scaffold isolated from *I. labyrinthus.* Such flexible biomaterial can be easily placed on the surface of human skin.

One possible application of bandage-like chitinous materials extracted from *I. labyrinthus* (Figure 11) may be hemostatic dressing for healing severe hemorrhagic wounds. Blood comprises a large amount of water, therefore the hemostatic materials should be highly absorbent and characterized by a high proportion of swelling fibril bundles, and present moisture wicking properties [64]. It has already been shown that chitin of crustacean origin induces blood coagulation by adhering to platelets, forming a chitin/platelet complex that promotes the polymerization of the fibrin monomer to form a blood clot (for review, see [65]). Additionally, chitin applied to wounds may attract histiocytes containing abundant lysozyme. As a result, at the early stage of wounds, the chitin dressing may induce fibroblasts to produce fine type III collagen through histiocytes [66]. In fact, several studies have been carried out on chitin in the form of nanocrystals [67] or powders [64] and non-woven fabrics [65] as a potential hemostatic biomaterial. To overcome the technical limitations associated with the transformation of chitin nanopowder to fibrillar material, we propose the scientific community evaluate the hemostatic properties in future clinical settings for 3D-structured sponge chitins, including those from *I. labyrinthus*.

**Figure 12:**
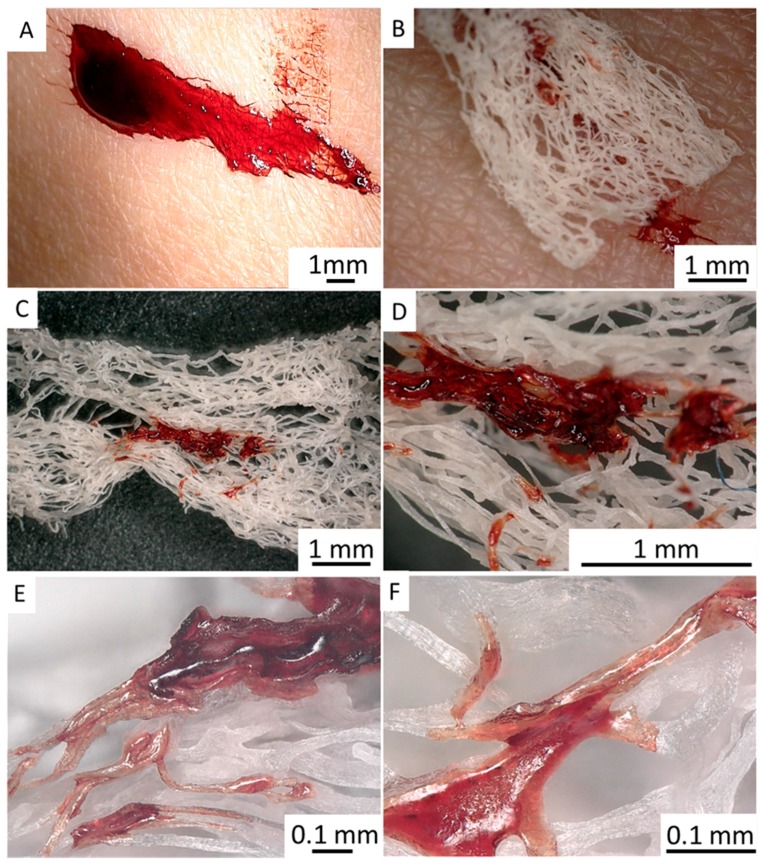
Chitinous scaffold isolated from *I. labyrinthus* can immediately absorb pig blood (**A**–**D**) from the skin surface. The blood is “sucked out” and located within the chitinous microtubes (**E**,**F**) in contrast to synthetic bandage (Figure 13), where blood is adsorbed on the surface and in the interspace of the monolithic, non-tubular microfibers. Pig blood (Südost-Fleisch GmbH, Altenburg, Germany).

**Figure 13:**
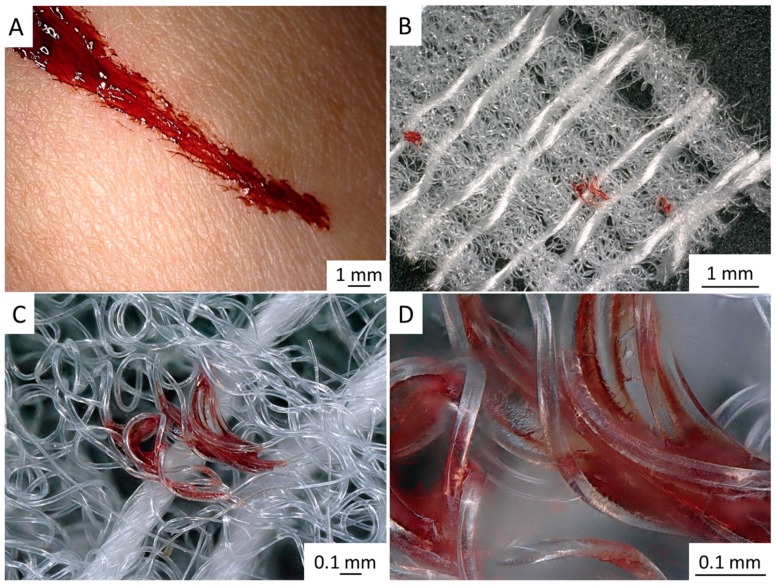
Synthetic bandage Aluderm^®^ (**A**,**B**) was used for blood absorption. Absorbed blood (**C**) was located on the surface of corresponding monolithic fibers, as well as between them (**D**).

Our first experiments (Figure 12) demonstrate that *I. labyrinthus* chitinous scaffold immediately absorbs blood of swine origin (obtained from a butcher shop) from the surface of skin (Figure 12A). In contrast to a similar experiment with synthetic gauze fabric (Figure 13), sponge chitin is able to absorb blood deep into the microtubes (Figure 12E,F) immediately due to excellent capillary effects. This suggests future investigation into the possible application of such ready-to-use chitinous constructs for storage of absorbed blood (including DNA) using corresponding cryopreservation techniques.

We suggest that the utilization of a naturally pre-structured fibrillar chitin scaffold of poriferan origin can be an important step of determining improved hemostatic dressings. Additional studies are required to determine the different outcomes that may result from their use, particularly in terms of potential adverse effects and safety. A sustained effort is needed to discover novel and safer hemostatics, mainly in countries in which there is limited access to the currently available agents [68]. This will require future comprehensive study, including the following steps: (i) Erythrocyte agglutination test; (ii) blood interaction test; (iii) sorption of blood; and (iv) complete blood count test. Such analyses could examine the effectiveness and the mechanism of sponge chitin interaction with human blood under in vitro and in vivo conditions [69]. Additionally, it is necessary to evaluate biological performance of sponge chitin-based absorbable hemostats, and to compare with reports for cellulose-based materials [70].

## 3. Materials and Methods

### 3.1. Location and Collection

The sponge *Ianthella labyrinthus* (WAM Z87073) was collected by J. Fromont and L. Kirkendale at station SOL47/W/A042 (15°36’46.10” S, 124°04’22.92” E to 15°36’44.77” S, 124°04’22.38” E), Kimberley, Western Australia in March 2015 at depth of 35.3–35.5 m. Morphological identification was supported by molecular barcoding and comparison against reference material of *I. labyrinthus* and other *Ianthella* spp. from the Western Australian Museum using the 28 S rRNA C-region barcoding for sponges. (see [71] for methodological details).

### 3.2. Isolation of Chitinous Skeleton from the Sponge and Identification of Selected Bromotyrosines

The procedure for isolation of chitin-based scaffolds from Ianthellidae sponges, as reported by us [30,72], was followed here. In brief, it involved the following steps: (i) Sponge skeleton (Figure 1A) was washed three times with distilled water for removal of water-soluble compounds, and then bromotyrosines were extracted with methanol (Figure 1B); (ii) residual fragments were placed into plastic boxes with 2.5 M NaOH at 37 °C for 72 h to remove cells, proteins, and pigments; (iii) samples were treated with 20% acetic acid at 37 °C for a period of 5–8 h to remove residual calcium carbonates, and then washed in distilled water up to pH 6.8. This isolation procedure was repeated three times to obtain colorless tubular scaffolds (Figure 4, Figure 5 and Figure 7). The purity of isolated chitin scaffolds has been proved according to standard analytical procedures as described previously [30].

The methanolic extracts of sponge fragments represented in Figure 1 were analyzed using a Shimadzu HPLC system, coupled to a UV-VIS detector (Shimadzu, USA; Waters SunFire Prep OBD C18 column (30 × 75 mm)). Routine detection was at 215 and 241 nm. A solvent system consisting of MeCN (A) and H_2_O (B) at a gradient increasing linearly from 0 to 100% was used for compound separation. LCMS analyses were carried out on an Agilent 1100 (Agilent, USA) LC system equipped with a G1956 MSD detector. Zorbax C18 RR column was used, and gradient elution with 0.1% HCOOH in H_2_O–MeCN was applied.

Identification of the substances was based on LCMS spectra and the data were compared with the literature. The compound was a simple bromotyrosine derivative with four bromines (m/z 698 (1), 700 (4), 702 (6), 704 (4), 706 (1)) and was consistent with a molecular formula C_21_H_23_Br_4_N_3_O_4_. These data suggest the compound was aplysamine 4 [73] or aplysamine 8 [74].

### 3.3. Stereomicroscopy Imaging

Photographic images were taken with a Keyence VHX-6000 digital optical microscope. We used pig blood (Südost-Fleisch GmbH, Altenburg, Germany) [8] and Bandage Aluderm^®^ (W. SÖHNGEN GmbH, Germany) gauze fabric for comparative experiments (see Figure 11 and Figure 12).

### 3.4. Differentiation and Culture of Human iPSC-CMs

The sponge chitinous scaffolds were prepared and autoclaved in PBS (Sigma-Aldrich, D8537-500 mL, St. Louis, Missouri, USA). Sterile scaffolds were cut in and pre-incubated either with the extracellular matrix mimetic Geltrex^®^ (Thermo Fisher Scientific, 2 mg/mL in DMEM, Waltham, MA, USA) or DMEM (Thermo Fisher Scientific, DMEM-F12 + l-Glutamine) as a control for 1.5 h at 37 °C. To obtain cardiomyocytes from iPSCs, cells were differentiated using the modulators CHIR99021 (day 0, start of differentiation) and IWP2 (day 2) according to established protocols [75,76]. iPSC-CMs differentiated from iPSCs lines created from two healthy patients (iBM76.3 and iWTD2.3) were tested in this study to account for variabilities in individual genetic background. After differentiation, iPSC-CMs were maintained in standard cardiomyocyte medium (RPMI1640 medium (Thermo Fisher Scientific) with 2% B27 supplement (Thermo Fisher Scientific) at 37 °C, 5% CO_2_. After one month of maturation (days 28–35), beating iPSC-CMs were detached using collagenase B (1 mg/ml) for 30–60 min at 37 °C and transferred into fresh reaction tubes. iPSC-CMs were singularized by digestion in trypsin/EDTA (Thermo Fisher Scientific, 0.25% Trypsin-EDTA) for 8 min at 37 °C. Subsequently, iPSC-CMs were resuspended in digestion medium (RPMI1640, 2% B27 supplement, 15% FBS, 2 µM thiazovivin) in a density of 3 million cells/mL. Sponge scaffolds were incubated in a volume of 150 µL iPSC-CM suspension overnight for 12–16 h at 37 °C. Afterwards, sponge scaffolds were carefully transferred to a transwell plate (Costar, 6 Transwell Well Plate, 24 mm Insert) prepared with 2.5 mL standard cardiomyocyte medium. iPSC-CMs on chitin scaffolds were cultured for 20 days with medium exchange every 2–3 days. Documentation of the cultures was performed using light microscopy (Axiovert100 equipped with Leica MC170 HD camera, Jena, Germany).

### 3.5. Immunostaining and Fluorescence Microscopy

After 20 days, iPSC-CM cultures were carefully washed 2 times with PBS (Sigma-Aldrich) and fixed in ice cold methanol (VWR, Radnor, PA, USA)–acetone (Merck, Darmstadt, Germany) solution (MeOH/Ac ratio 7:3 *v*/*v*) for 10 min at –20 °C. Fixed samples were washed 3 times with PBS at room temperature (RT) and blocked in PBS with 1% BSA (Sigma-Aldrich) and 0.1% TritonX-100 (Ferak) overnight. Samples were transferred to a 35 mm dish with glass bottom (MatTek, Ashland, MA, USA). Samples were incubated in primary antibody solution containing mouse anti-α-actinin (Sigma-Aldrich, A7811-100UL, 1:500) and rabbit anti Ki-67 (Abcam, ab833, 1:400, Cambridge, UK) in PBS with 1% BSA overnight at 4 °C. Afterwards, samples were washed 3 times with PBS at RT and incubated with a second antibody solution containing AlexaFluor488-labeled goat anti-mouse (Thermo Fisher Scientific, A11001, 1:1000, Waltham, MA, USA) and AlexaFluor546-labeled goat anti-rabbit (Life technologies, A11035, 1:1000) antibody conjugates for 1 h at RT. Cell nuclei were stained with Hoechst33342 (molecular probes, H3570, 1:800) for 10 min at RT. Stained samples were washed 3 times with PBS, prepared in Flouromount-G (SouthernBiotech, Birmingham, AL, USA), and stored at 4 °C until imaging. Negative controls (incubation with secondary antibody only) were performed to evaluate the specificity of the staining. Overview pictures of chitin scaffolds were observed with a Keyence BZ-X710 imaging platform and multi-dimensional capture mode (XY-Stitching, phase contrast and Hoechst33342 channel). Fluorescence images to characterize iPSC-CM attachment to the scaffolds and to quantify the population of Ki-67 positive cells were obtained with a confocal laser scan microscope (LSM880) at the imaging facility of TU Dresden. To create the images of specific regions, z-stacking of 3–5 images was captured and analyzed (2.5–10 µm z-range) using ImageJ software (sum/max of intensities). Brightness and contrast were processed for each individual image. Relative quantification of Ki-67 positive cells was performed by manual counting of at least 300 cells from 3D reconstructions of different regions of each sample using ImageJ.

## 4. Conclusions

Industrially obtained chitin of crustaceans or fungal origin requires numerous unit operations and technological processes to transform it from powders or flakes into sponge-like materials or foams. It is technologically difficult and as a result, it is not economically feasible or ecologically friendly. The isolation of chitinous 3D naturally pre-designed scaffolds from marine sponges opens the gate to overcome these difficulties. Chitin of poriferan origin has been recently recognized as a renewable source of unique naturally prefabricated 3D constructs. Diversity in size, shape, and porosity of such chitin is based on structural peculiarities of the original sponge skeletons. Ianthellids can obtain good growth under marine farming conditions due to special ability to regenerate their chitin-based skeletal structures very quickly (12 cm in year [43]). Additionally, it has been proven that chitinous scaffolds of poriferan origin possess tubular structure and remarkable swelling properties [8], due to capillary forces and the porosity of these materials, which evolved due to millions of years and are evolutionarily optimized to support the cell growth and proliferation. Due to high thermal stability [16,18,49], chitin-based materials can be sterilized by autoclaving at 121 °C [77]. Here, we show that the demosponge *I. labyrinthus* has remarkable biomimetic potentialities, as a source of natural compounds useful for pharmaceutical industry and of new biomaterials for tissue engineering and regenerative medicine, simultaneously. In this work, we have demonstrated that the chitin-based skeleton, after bromotyrosines extraction, is a suitable scaffold for iPSC-CM cultivation. The peculiar microtubular organization of the *I. labyrinthus* skeleton is also able to absorb water and blood, suggesting a biomimetic approach for new generation of hemostats, with higher performances compared to the cellulose-based ones traditionally used in clinics.

## Supplementary Materials

Supplementary materials can be found at https://www.mdpi.com/1422-0067/20/20/5105/s1; Supplementary Video 1: iPSC-derived cardiomyocytes on Geltrex-coated (left) and uncoated (right) chitin scaffolds of *I. labyrinthus*. Cells on chitin scaffolds were cultured in transwell plates with low medium levels for 20 days. Videos were obtained using phase contrast microscopy (30 fps); Supplementary Video 2: 3D reconstruction of iPSC-CM layer on *I. labyrinthus* chitin scaffolds from fluorescence microscopy images. Colors represent α-actinin (green) and Ki-67 (magenta) and cell nuclei (blue). Based on the 3D reconstructions, colocalization of Ki-67 and nuclei was determined to quantify cell proliferation.
